# Effects of seasonal temperature regimes on embryo growth and endogenous hormones of *Taxus chinensis* var. *mairei* seeds

**DOI:** 10.3389/fpls.2023.1114629

**Published:** 2023-03-03

**Authors:** Man Zhou, Yan Xu, Fang Wang, Xuejun Yang, Shunbao Lu, Yanjie Zhang

**Affiliations:** ^1^ College of Life Sciences, Jiangxi Normal University, Nanchang, China; ^2^ State Key Laboratory of Vegetation and Environmental Change, Institute of Botany, Chinese Academy of Sciences, Beijing, China

**Keywords:** *Taxus chinensis* var. *mairei*, endogenous hormones, embryo length, E : S ratio, split rate

## Abstract

Seed dormancy is a mechanism that prevents seeds from germinating at times of the year when conditions are unfavorable, that is, when the chance of seed survival is low. Determining the seasonal dynamics of seed dormancy is important for exploring how plant regeneration is adapted to the environment. We studied the seed dormancy status of *Taxus chinensis* var. *mairei*, an endangered species in China, under simulated seasonal temperature regimes. The embryo length, embryo-to-seed (E : S) ratio, and percentage of seeds with a split seed coat increased when seeds were stratified at spring and autumn temperature regimes. The abscisic acid (ABA) content decreased during stratification at simulated seasonal temperatures, but no obvious pattern in the content of gibberellic acid (GA) and indole acetic acid (IAA) was observed. The GA–ABA and IAA–ABA ratios increased during stratification. These results suggest that *T. chinensis* var. *mairei* seeds have morphophysiological dormancy, and that the seasonal dynamics of seed dormancy break are controlled by endogenous hormones and their balances, which was confirmed by the results of a field experiment. Our study provides useful information for understanding the natural population regeneration and propagation of this threatened species.

## Introduction

Seed dormancy can prevent germination when the environment is unfavorable for seedling establishment and survival (Baskin and Baskin, 2014), and it is broken in response to changes in seasonal environmental conditions in the habitat, which in turn help to control the time of germination (Linkies et al., 2010; Song et al., 2018). There are five classes of seed dormancy: physical dormancy (PY), morphological dormancy (MD), physiological dormancy (PD), morphophysiological dormancy (MPD), and combinational dormancy (PY + PD) (Baskin and Baskin, 2004). Embryos of MD seeds are underdeveloped and require time to grow inside the seed before germination. Embryos in seeds with PD are fully developed but require dormancy-breaking treatment before they can germinate. PD is widely found in the seeds of gymnosperms and most angiosperms, and is the most common class of dormancy. MPD is a combination of MD and PD. Seeds with MPD exhibit both underdeveloped and physiologically dormant embryos. MPD can be divided into two levels and nine types depending on the conditions required to break PD and to promote embryo growth (Baskin and Baskin, 1990; Baskin and Baskin, 2004; Baskin and Baskin, 2014; Rhie et al., 2016; Lee et al., 2018).

Seed dormancy and germination are regulated by endogenous plant hormones, including abscisic acid (ABA), gibberellins (e.g., gibberellic acid, GA), and indole-3-acetic acid (IAA) (Miransari & Smith, 2014). ABA is the main hormone involved in the initiation and maintenance of dormancy in plants (Hilhorst, 1995; Nambara and Marion-Poll, 2003; Kucera et al., 2005). For example, in *Arabidopsis thaliana* seeds, ABA levels are higher in dormant seeds than in non-dormant seeds (Ali-Rachedi et al., 2004). In addition, the overexpression of *ABA* mRNA in *Nicotiana plumbaginifolia* cv. Viviani increases ABA abundance and delayed germination, whereas the expression of antisense *ABA* mRNA decreased ABA levels and promotes germination (Frey et al., 1999). The application of exogenous ABA inhibits seed germination (Chauffour et al., 2019); however, fluridone, an ABA biosynthesis inhibitor, effectively breaks the seed dormancy of *Phellodendron amurense* var. *wilsonii* (Chen et al., 2009). In addition, GA is a positive regulator in the process of seed germination (Kucera et al., 2005). It has been reported that GA promotes seed germination, but is not involved in the seed dormancy release of *Avena fatua*. (Fennimore and Foley, 1998). The role of IAA in seed germination is controversial. IAA inhibits seed germination in *Triticum aestivum* (Ramaih et al., 2003); however, IAA synthesis increases during germination of *Phaseolus vulgaris* seeds (Bialek et al., 1992). ABA and GA play antagonistic roles in the initiation, maintenance, and termination of seed dormancy (Amen, 1968; Wareing and Saunders, 1971). Experiments in sorghum and maize have demonstrated that ABA and GA simultaneously regulate seed dormancy and germination (Steinbach et al., 1997; White and Rivin, 2000; White et al., 2000). Seed dormancy release is associated with ABA degradation and GA biosynthesis, which result in a low ABA–GA ratio (Finch-Savage and Leubner-Metzger, 2006). Similar conclusions were reported in a study of *Musella lasiocarpa* seeds (Tang, 2014a).

Seasonal variation in temperature is a major environmental factor affecting seed dormancy (Hilhorst, 1998; Benech-Arnold et al., 2000; Brändel, 2004). The dormancy state of the seeds of Arabidopsis thaliana, i.e. the primary dormancy and secondary dormancy, is correlated with seasonal changes, which stops until environmental conditions are conducive to seedling formation (Ibarra et al. 2016). In summer annual plants, seed dormancy is released and induced by low winter temperatures and high summer temperatures, respectively (Brändel, 2004). After PD is broken by winter temperatures, some *Pinus koraiensis* seeds germinate in the first year after embryos grow to a sufficient length (i.e., from April to June), whereas the majority of the seeds enter secondary dormancy in the summer and germinate in the second spring after PD is broken (Song et al., 2018). In temperate regions, seed germination in many species requires exposure to summer and winter temperatures to break dormancy (Baskin and Baskin, 2003).

*Taxus chinensis* var. *mairei* is an evergreen tree that is a unique tertiary relic in China. In addition to being ornamental, it is an important source of timber and has medicinal uses (Zhang et al., 2012; Xu et al., 2022). This species is distributed sporadically in the south of the Qinling Mountains and north of the Lingnan Mountains, China, growing in humid areas with favorable hydrothermal environments (Xie, 2013; OU, 2022). Given that taxol, an anticancer drug, is obtained from the root, stem, leaf, and bark of *Taxus*, interest in the *Taxus* genus has increased (Vidensek et al., 1990; Witherup et al., 1990). *T. chinensis* var. *mairei* has been listed as an endangered species because of both the destruction of its natural habitat and low natural reproduction due to deep seed dormancy (Zhu et al., 1999). The seeds of this species are dispersed in winter and their dormancy is broken only after a long period; germination then occurs in the spring of the third year after dormancy is broken. Seed germination and seedling survival percentages are low, which has resulted in the population decline of this species in natural conditions (Zhang et al., 2012; Möller et al., 2020).

Warm stratification and cold stratification are commonly used methods for simulating seasonal temperature regimes in the natural environments of temperate zones (Chien et al., 1998; Baskin and Baskin, 2004). The effect of combining warm stratification and cold stratification on the dormancy breaking of *T. mairei* seeds has already been reported (Chien et al., 1995). However, seeds of the same species that are collected from different regions can still respond differently to seasonal temperatures. For example, the germination percentages of *Ilex maximowicziana* seeds that were collected from tropical environments were higher at high temperatures than the germination percentages of those that were collected from subtropical environments (Chien et al., 2011). Therefore, a deeper understanding of the dormancy status of *T. chinensis* var. *mairei* under local temperature regimes is needed to reveal the general adaptive ability of this species to its local environment. In addition, no study has been conducted that examines the seed dormancy dynamics of this species under seasonal temperature regimes.

To understand the seasonal seed dormancy dynamics of the *T. chinensis* var. *mairei* species, we studied the embryo growth rate and hormone contents of the seeds under simulated seasonal temperature regimes. The specific aims of this study were to (1) determine the class of dormancy, (2) evaluate the effect of stratification at seasonal temperature regimes on embryo growth rate and seed germination, and (3) identify the role of endogenous hormones in the seed dormancy of *T. chinensis* var. *mairei*. Knowledge of the seasonal dynamics of dormancy status and the changes in endogenous phytohormone levels can provide a mechanistic understanding of seed dormancy in *T. chinensis* var. *mairei*, which may in turn lead to the design of more effective preservation strategies for this endangered species.

## Materials and methods

### Seed collection

*T. chinensis* var. *mairei* seeds were collected from a natural population growing in the valley of an evergreen deciduous broadleaf mixed forest at an altitude of 400–500 m.a.s.l. (meters above sea level) in Xiushui County (114°32′47.88″E, 29°01′34.60″N), Jiangxi Province, China, in December 2016. The experiment was conducted in May 2017. The *T. chinensis* var. *mairei* seed population collected was aged 20–40 years. In the laboratory, seeds were soaked in water and scrubbed manually to remove red arils. Thereafter, seeds were air dried and stored at room temperature (i.e., at 25°). The size and weight of seeds were measured. Seed water content was determined by the oven-drying method (i.e., 130 ± 2° for 24 h), and viability was determined using the threshold of toxicological concern (TTC) method (Yan, 2001).

### Germination of fresh seeds

Seeds were sterilized with 0.1% potassium permanganate (KMnO_4_) solution for 30 minutes and then rinsed in running water for 3 hours. Subsequently, seeds were mixed with wet sterile sand at a ratio of 1 : 3 and placed in Petri dishes (9 cm) in an incubator at 25°. Seeds were exposed to a light (400–700 nm) regime of 3,000 lx for 12 h each day. Three replicates of 50 seeds each were monitored for 40 days to observe germination.

### Water imbibition of seeds

Three replications of 30 non-scarified and 30 scarified seeds each were weighed with a digital balance, and the initial fresh weights of seeds were recorded. The seeds were submerged in distilled water in a 25° incubator. The seeds were removed periodically (i.e., at 3-hour intervals during the day and at 12-hour intervals at night for the first 2 days, and at 12-hour intervals thereafter) from the water, dried with filter paper, weighed, and then returned to the water. The experiment ended when an increase in seed weights was no longer observed. The water imbibition percentage was calculated using the following formula:

Water imbibition


$$percentage\;=\frac{Weight\;of\;imbided\;seed-Initial\;weight\;of\;seed}{Initial\;weight\;of\;seed}\;\times \;100$$

### Stratification at seasonal temperature regimes

To determine the response of seeds to seasonal temperature regimes, four temperature regimes were used to simulate local (i.e., Xiushui County, Jiangxi Province, China) seasonal temperatures, i.e., a high of 22.1° and low of 12.7° to simulate spring, 32.4°/23.5° for summer, 23.9°/14.3° for autumn, and 11.0°/2.8° for winter. Seeds were alternately exposed to high and low temperatures for 12 h each day. These seasonal temperature regimes were calculated from local average maximum and minimum air temperatures that were provided by the Jiangxi Meteorological Bureau, Jiangxi Province, China. Seeds were mixed with wet sand [16% moisture at a ratio of 1 : 3 and placed in a plastic box (19 cm length × 13 cm width × 12 cm height] in an incubator. Each plastic box contained 600 seeds. There were three boxes (i.e., replicates) at each temperature regime. The boxes were observed weekly, and the sand was kept moist with the addition of distilled water. Seeds were stratified at each temperature regime for 12 months, and 150 seeds were randomly sampled every 3 months for seed embryo observation and hormone concentration determination.

### Determination of embryo growth and seed germination

For embryo length measurement, 20 seeds were randomly chosen and dissected under a microscope using a scalpel, and their embryo lengths (E) and seed lengths (S) were measured using an ocular micrometer. The E : S ratio was then calculated. The number of seeds with a split seed coat, which indicated that the seeds were starting to germinate, was recorded.

### Determination of endogenous hormone concentration

Seeds used for the determination of endogenous hormone were immediately frozen after sampling and then stored at −80° until analysis. Three replicates of this experiment were performed, and each replicate included 0.3 g of seeds. Samples were extracted with 2 mL of 80% (v/v; volume/volume) methanol containing 1 mmol/L butylated hydroxytoluene (BHT) in an ice-cooled mortar, ground into a homogenate, and then transferred to a 10-mL centrifuge tube. The mortar was washed with 2 mL of sample extraction buffer again and transferred to the same tube. The extract was incubated at 4° for 4 h and centrifuged at 4,000 rpm for 15 min at the same temperature. The supernatant was collected and passed through a Sep-Pak^®^ C18 cartridge (Waters Corporation, Milford, MA, USA) based on the following procedure: balance column with 1 mL of 80% methanol → load samples → collect samples → wash column with 5 mL of 100% methanol → elute hormones with 100% ethyl ether (5 mL) → 100% methanol (5 mL)  → recycle. The samples were then transferred into a 5-mL plastic centrifuge tube and concentrated in a vacuum to remove the methanol. The volume was increased to 1 mL using 0.01 mol/L phosphate-buffered solution (pH 7.4). The concentrations of ABA, GA, and IAA were determined using an enzyme-linked immunosorbent assay (ELISA) (Wu et al., 1988). The optical density (OD)value at 490 nm of each sample was determined using a microplate reader (RT-6100 Microplate Reader; Rayto Life and Analytical Sciences Co., Ltd, Shenzhen, China).

### Seasonal seed dormancy dynamics in the field

The field experiment was conducted at Jiangxi Normal University, Nanchang, Jiangxi Province, China (135 km from the natural population), with similar climatic conditions as those of the seed collection site. Seeds were placed into nylon bags and buried in the soil at a depth of 3 cm. Each bag contained 1,000 seeds, and three replicate pots were placed in a shady forest. Two hundred seeds were collected from each bag every 3 months from 20 May 2017 to 20 May 2018. Seed embryo observation and hormone content determination were conducted using the methods described above.

### Data analyses

The means and standard errors were calculated for embryo length, E : S ratio, percentage of seeds with a split seed coat, phytohormone contents, the ratio of hormones, and field germination percentage. Repeated measures analysis of variance (ANOVA) was used to determine the effects of temperature regime and time on the embryo length, E : S ratio, percentage of seeds with a split seed coat, hormone contents, and hormone ratios. Data were tested for assumptions of normality and homogeneity of variance prior to repeated measures ANOVA. Embryo length data were square root-transformed. The E : S and hormone ratios were arcsine transformed. Data were analyzed using IBM SPSS^®^ Statistics version 20.0 (IBM Corporation, Armonk, NY, USA) software at a p-value of 0.05.

## Results

### Germination and water uptake of fresh seeds

**Figure 1:**
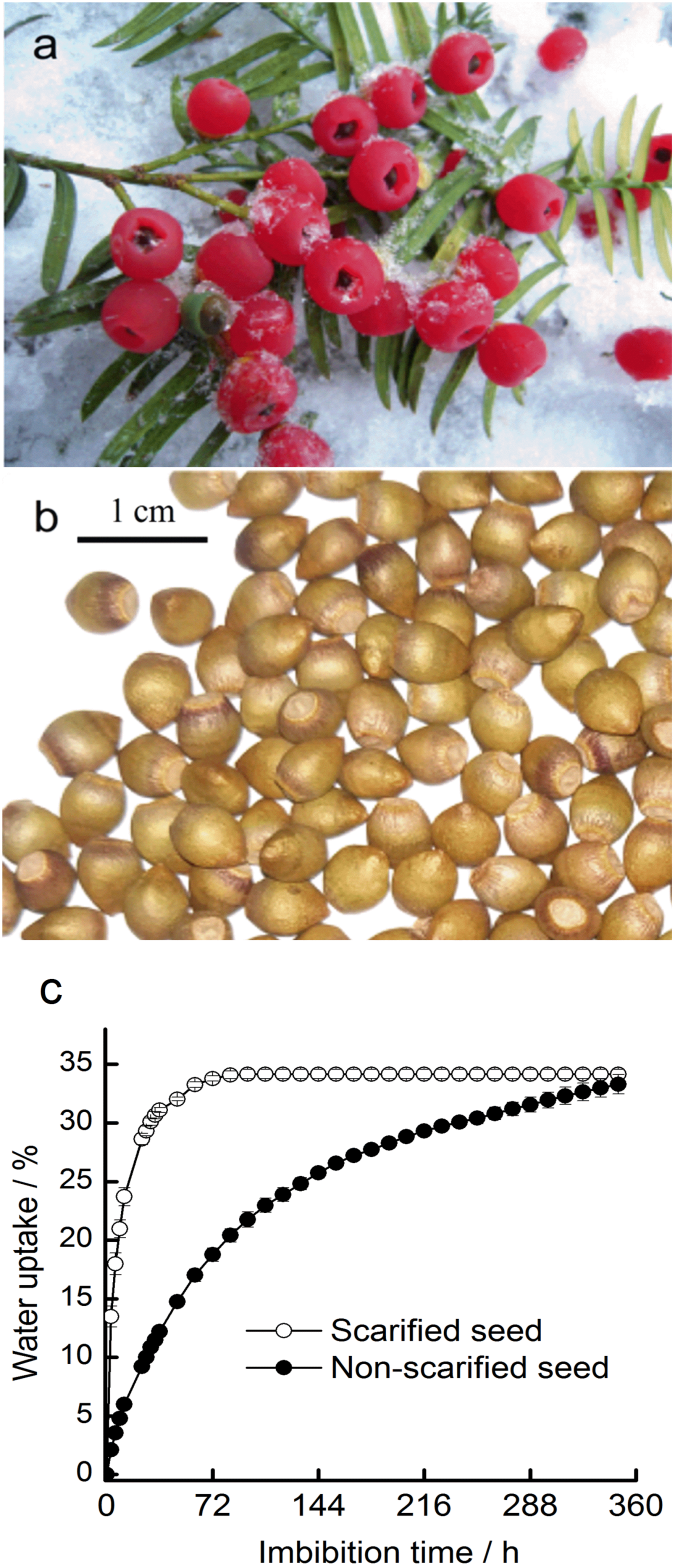
The fruits **(A)**, seeds **(B)**, and seed water uptake **(C)** of *Taxus chinensis* var. *mairei*.

The aril of *T. chinensis* var. *mairei* was red (**Figure 1A**). The episperm was hard and was either yellowish or brown in color. The hilum was elliptical or triangular in shape (**Figure 1B**). The long axis of seeds was 6.55 ± 0.32 mm. The transverse diameter was 5.08 ± 0.12 mm, and the longitudinal diameter was 4.24 ± 0.12 mm. The 1,000 seed weight was 65.34 g, and the water content of the seeds was 5.97%. The seed viability was 77%.

After 40 days of stratification, none of the fresh seeds had germinated.

The water uptake of scarified seeds was initially increased compared with non-scarified seeds, but the two types of all seeds exhibited a similar rate of water uptake at 324 h (**Figure 1C**). The rate of water uptake of scarified seeds plateaued after exposure to water for 96 h, whereas that of non-scarified seeds plateaued after exposure to water for 324 h.

### Embryo growth rates at different simulated seasonal temperature regimes

**Figure 2:**
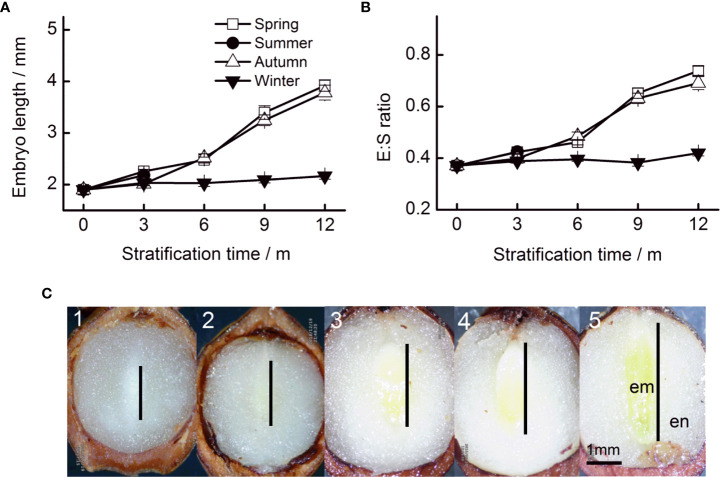
Embryo length **(A)** and the E : S ratio **(B)** of Taxus chinensis var. mairei seeds under stratification at four seasonal temperature regimes. **(C)** Embryo growth at spring temperature regimes (1, before stratification; 2, after 3 months; 3, after 6 months; 4, after 9 months; and 5, after 12 months). The vertical bars represent standard errors of the mean (n = 3). E : S ratio, embryo : seed ratio.

The initial embryo length was 1.90 mm (**Figure 2A**). Embryo length reached 3.92, 3.78, and 2.16 mm when stratified for 12 months at simulated spring, autumn, and winter temperature regimes, respectively. Seasonal temperature, time, and their interactions had significant effects on embryo length (**Table 1**). The embryos of seeds stratified at summer temperatures grew during the first 3 months, but thereafter, seeds decayed (**Figure 2A**). The fastest embryo growth rate was observed when seeds were stratified at spring and autumn temperatures. However, in the first 3 months, embryos grew at a faster rate at spring than at autumn temperatures. During stratification at winter temperatures, embryos grew slowly.

**Table 1:** Effects of seasonal temperature regimes and time on embryo growth and endogenous hormones in *Taxus chinensis* var. *mairei* seeds.

Factor	Temperature		Time		Temperature × time	
	°F	*p*-value	°F	*p*-value	°F	*p*-value
Embryo length	126.238	0.000***	183.605	0.000***	31.665	0.000***
E : S ratio	132.514	0.000***	152.793	0.000***	28.075	0.000***
Seed split rate	87.587	0.000***	402.233	0.000***	32.745	0.000***
ABA content	17.696	0.003**	183.190	0.000***	4.708	0.001**
GA content	1.057	0.331	6.495	0.001**	3.321	0.015*
IAA content	6.033	0.037*	5.360	0.003**	2.006	0.090
GA–ABA ratio	13.481	0.002**	42.378	0.000***	6.446	0.000***
IAA–ABA ratio	4.918	0.032*	93.743	0.000***	7.434	0.000***

ABA, abscisic acid; GA, gibberellic acid; E : S ratio, embryo-to-seed ratio; IAA, indole acetic acid. *, **, and *** indicate P ≤ .05, ≤.01, and ≤.001, respectively.

The pattern of change in the E : S ratio was similar to that of embryo growth rates, that is seasonal temperature regime, time, and their interactions had significant effects on the E : S ratio (**Table 1**). The E : S ratio increased fastest when seeds were stratified at spring and autumn temperatures. The E : S ratio increased slowly at winter temperatures (**Figure 2B**).

The color of cotyledons changed obviously during stratification. Before stratification, the entire embryo was ivory-white. However, the cotyledons turned green after 6 months of stratification at spring and autumn temperatures (**Figure 2C**).

**Figure 3:**
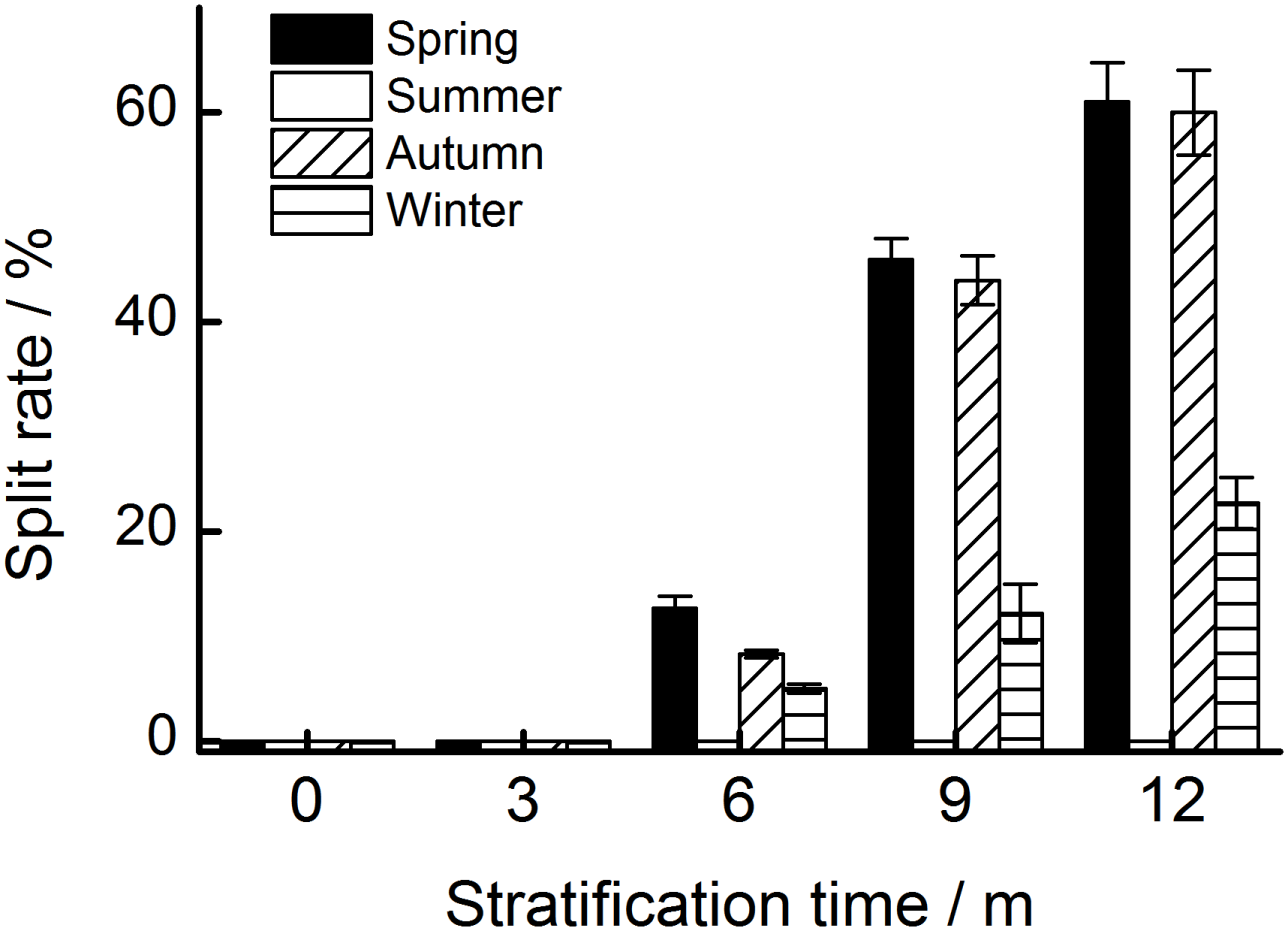
The percentage of seeds with a split seed coat stratified at four seasonal temperature regimes. The vertical bars represent standard errors of the mean (n = 3).

After 12 months, 60.0%, 61.0%, and 22.8% of the seeds stratified at spring, summer, and autumn temperatures, respectively, had a split coat (**Figure 2C**). Seasonal temperature, time, and their interactions had significant effects on the percentage of seeds with a split coat (**Table 1**). The percentage of seeds with split coats was highest when seeds were stratified at spring and autumn temperatures, and lowest when they were stratified at winter temperatures (**Figure 3**).

### Effects of stratification temperature on endogenous hormone levels in seeds

**Figure 4:**
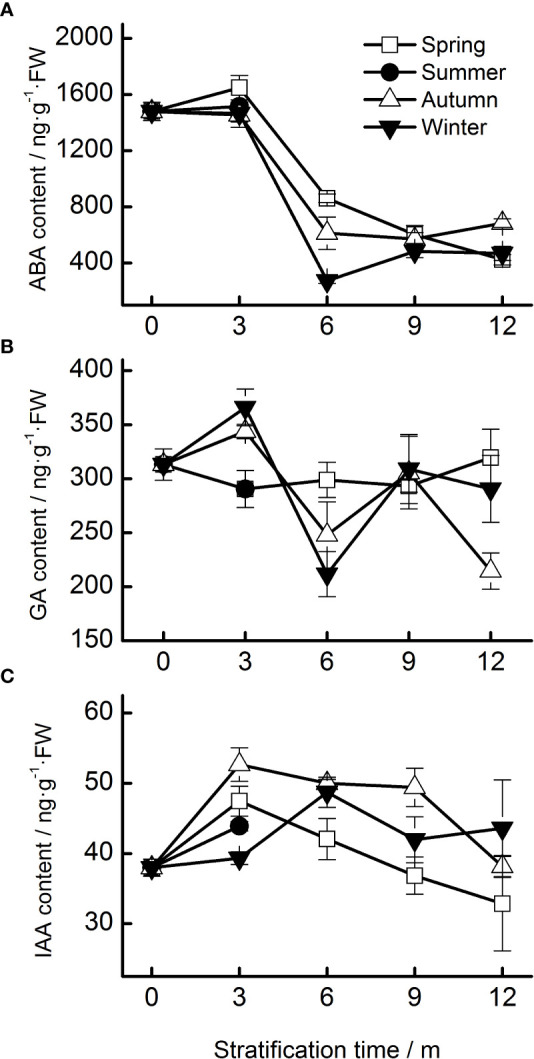
Endogenous hormone contents (**A**, ABA; **B**, GA; **C**, IAA) of Taxus chinensis var. mairei seeds under stratification at four seasonal temperature regimes. The vertical bars represent standard errors of the mean (n = 3). ABA, abscisic acid; GA, gibberellic acid; IAA, indole acetic acid.

The ABA content of seeds progressively decreased during the 12 months of stratification (specifically, during the spring, autumn, and winter temperature regimes. Seasonal temperature, time, and their interactions had significant effects on ABA content (**Table 1**). ABA levels in seeds stratified at spring, autumn, and winter temperatures were stable for the first 3 months but decreased rapidly afterwards (**Figure 4A**).

No obvious change in the content of GA was observed during stratification. The content of GA in seeds stratified at autumn and winter temperatures increased during the first 3 months and then decreased (**Figure 4B**).

Seasonal temperature, time, and their interactions exhibited significant effects on the IAA levels of the seeds (**Table 1**). The IAA content increased in the first 3 months of stratification at spring and autumn temperatures and then decreased (**Figure 4C**).

### Effects of stratification temperature on the ratios of endogenous hormones in seeds

**Figure 5:**
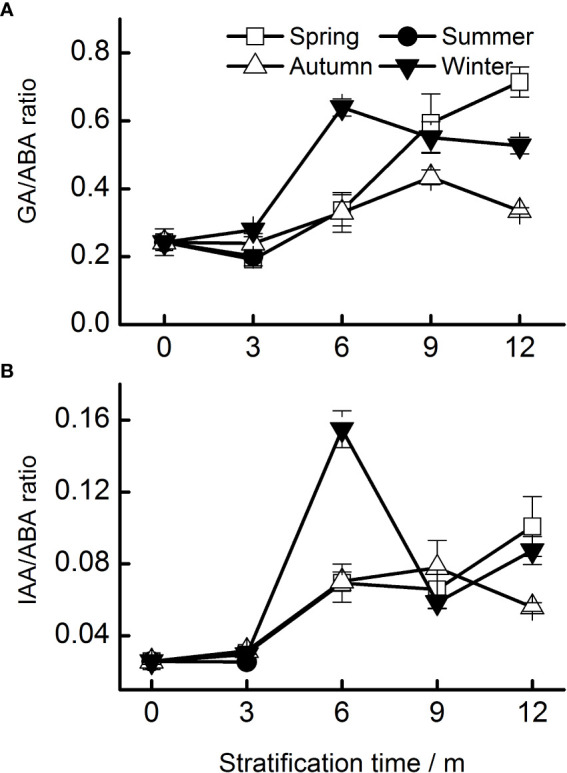
Ratios of hormones (**A**, GA–ABA; **B**, IAA–ABA) of Taxus chinensis var. mairei seeds stratified at four seasonal temperature regimes. The vertical bars represent standard errors of the mean (n = 3). ABA, abscisic acid; GA, gibberellic acid; IAA, indole acetic acid.

The IAA–ABA ratio was lower than the GA–ABA ratio (**Figure 5**). The GA–ABA and IAA–ABA ratios in all seeds increased significantly after 12 months of stratification. The highest GA–ABA ratio in seeds stratified at spring temperatures occurred after 12 months. The pattern of the IAA–ABA ratio was similar to that of the GA–ABA ratio.

### Relationship between embryo growth and endogenous hormones

The correlations between the embryo length, E : S ratio, and the percentage of seeds with a split seed coat were significant (*p*< 0.05; **Table 2**). The correlations between the percentage of seeds with a split coat and IAA content, GA–ABA ratio, and IAA–ABA ratio were also significant (*p*< 0.05). There were negative correlations between the ABA content, embryo length, E : S ratio, and the percentage of seeds with a split coat, indicating that ABA inhibited the growth of the embryo and seed germination. There were positive correlations between the GA–ABA ratio and embryo length, E : S ratio, and the percentage of seeds with a split coat, indicating that a high GA–ABA ratio promotes embryo growth and seed germination.

**Table 2:** Correlation analysis of the embryo growth and endogenous hormones in *Taxus chinensis* var. *mairei* seeds.

	Embryo length	E : S ratio	Seed split rate	ABA content	GA content	IAA–content	GA–ABA ratio	IA–ABA ratio
Embryo length	1							
E : S ratio	0.987**	1						
Seed split rate	0.933**	0.944**	1					
ABA content	–0.453**	–0.457**	–0.585**	1				
GA content	–0.189	–0.170	–0.180	0.363*	1			
IAA content	–0.376*	–0.348*	–0.355*	0.073	–0.074	1		
GA–ABA ratio	0.391*	0.411**	0.541**	–0.836**	–0.179	–0.257	1	
IAA–ABA ratio	0.225	0.249	0.317*	–0.793**	–0.363*	0.056	0.738**	1

ABA, abscisic acid; GA, gibberellic acid; E : S ratio, embryo-to-seed ratio; IAA, indole acetic acid. * and ** indicate P ≤ .05, ≤.01, respectively.

### Seasonal seed dormancy dynamics in the field experiments

**Figure 6:**
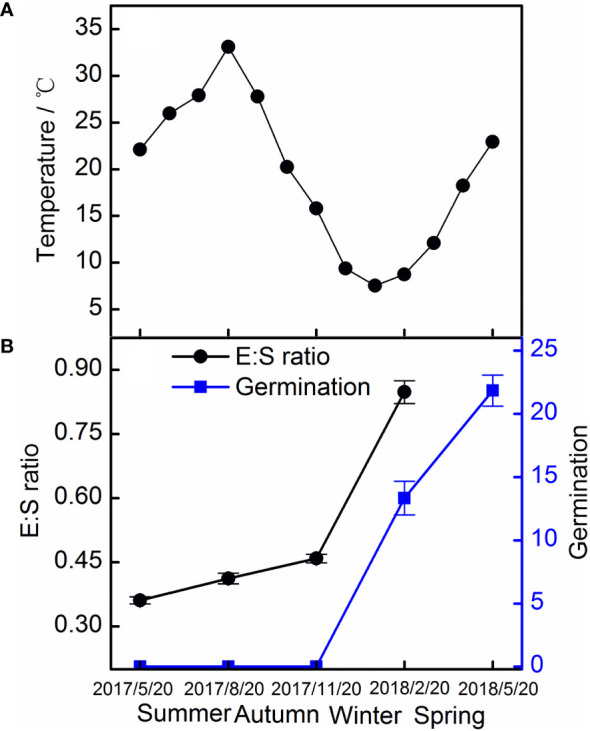
Monthly mean temperature **(A)** and the E : S ratio and germination of Taxus chinensis var. mairei seeds **(B)** in the field experiment. The vertical bars represent standard errors of the mean (n = 3). E : S ratio, embryo-to-seed ratio.

The E : S ratio of seeds in the field experiments increased slowly in the first 6 months, and then increased rapidly, reaching 84.82% after 9 months. Seeds started to germinate in February 2018, and germination reached 21.8% in May 2018 (**Figure 6**).

**Figure 7:**
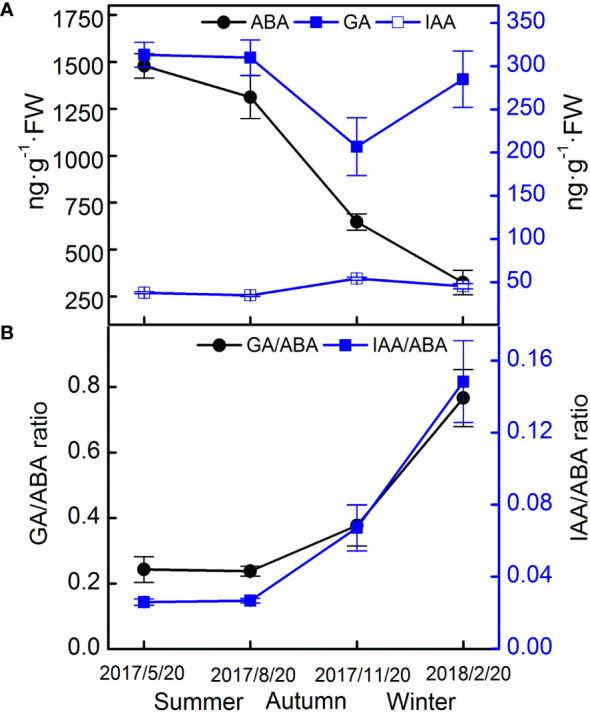
Endogenous hormone contents **(A)** and their ratios **(B)** of Taxus chinensis var. mairei seeds in the field experiment. The vertical bars represent standard errors of the mean (n = 3).

In the field experiment, the ABA levels decreased in seeds, but no obvious pattern in the levels of GA and IAA was observed (**Figure 7A**). The GA–ABA and IAA–ABA ratios increased rapidly from August 2017 to February 2018 (**Figure 7B**).

## Discussion

The initial viability of *T. chinensis* var. *mairei* seeds was high. However, in the germination test, none of the seeds had germinated after 40 days of incubation, indicating that the seeds were dormant. The seed coat of *T. chinensis* var. *mairei* was water permeable (**Figure 1C**); thus, the seed dormancy is not PY. The embryo was immature when the seeds matured and grew in our stratification experiments (**Figure 2C**), which suggests that the seeds had underdeveloped embryos. The ABA content decreased in both our stratification and field experiments, indicating in turn that *T. chinensis* var. *mairei* seeds exhibit PD (**Figures 4A**, **7A**). In addition, treatment with the exogenous gibberellin GA_3_ is effective at promoting seed germination (Zhang, 2007).

The values for embryo length, E : S ratio, and the percentage of seeds with a split coat when seeds were stratified at spring and autumn temperatures were higher than those when they were stratified at winter temperatures (**Figures 2**, **3**). This indicates that the embryo growth of *T. chinensis* var. *mairei* seeds requires warm temperatures. Our results are consistent with those of previous studies, which indicate that the embryos of many seeds with MPD need to grow at warm temperatures, followed by cold stratification conditions (Baskin and Baskin, 2004; Tang, 2014b; Li et al., 2017). For example, the embryo development of *Paris polyphylla* var. *chinensis* seeds is accelerated after 40 days of warm stratification (Li et al., 2015). Similarly, the E : S ratio of Korean pine seeds does not change during cold stratification but increases following warm stratification (Asakawa, 1955).

In our field experiment, *T. chinensis* var. *mairei* seeds only began to germinate after a combination of 3 months of high summer temperatures (May 2017–August 2017), 3 months of warm autumn temperatures (August 2017–November 2017), and 3 months of low winter temperatures (December 2017–February 2018). These results agree with those of Zhang et al. (2011), who found that the dormancy break and germination in this taxon are broken by warm, followed by cold stratification conditions. Therefore, warm autumn temperatures can release MD, and chill winter temperatures break PD. Then, the seeds germinate in the warm spring, which is favorable for seedling establishment. Such seasonal dormancy dynamics have also been reported in several species with MPD seeds. For example, seeds of *Jeffersonia diphylla* require a sequence of summer, autumn, and winter temperatures to break dormancy and germinate in the following spring (Baskin and Baskin, 1989). Although the seeds of *P. polyphylla* var. *yunnanensis* break their MD after 180 days of warm stratification, they germinate only after stratification at low temperatures (Huang et al., 2008). Stratification at neither high nor low temperature alone was found to break the dormancy of *Acanthopanax sessiliflorus* seeds, but exposure to cold temperature after warm temperature effectively released seed dormancy (Ge et al., 2007).

Abscisic acid (ABA) plays critical roles in regulating embryo development and seed germination (Bewley and Black, 1985; Bian et al., 2018). In our study, the ABA content of *T. chinensis* var. *mairei* seeds decreased after 6 months of incubation (**Figure 4**), and the field experiment produced similar results (**Figure 7A**). High ABA levels maintain seed dormancy and inhibit embryo growth (Kucera et al., 2005). ABA content typically decreases as seed dormancy is released. For example, during cold stratification or fluctuating temperature stratification, the ABA levels in *Myrica rubra* seeds significantly decreased (Chen et al., 2008), whereas during warm stratification, the ABA levels in embryos of *M. lasiocarpa* seeds continuously decreased (Tang, 2014a). After cold stratification, ABA levels in fresh *Acer morrisonense* seeds decrease to those that are similar to levels in germinated seeds (Chen et al., 2015). Our results confirm that ABA plays important roles in the regulation of dormancy status of *T. chinensis* var. *mairei* seeds.

No obvious pattern in the content of GA was observed during incubation (**Figure 4B**), indicating that GA did not play a major role in regulating the dormancy of *T. chinensis* var. *mairei* in seeds in the field experiments. The results are consistent with those from a study of *Hordeum vulgare* seeds, in which GA had no causal relationship with the loss of seed dormancy but seemed to be related to seed germination (Jacobsen et al., 2002). The biosynthesis of GA is necessary for the germination of non-dormant seeds (Hu et al., 2012). GAs levels in *P. amurense* var. *wilsonii* seeds increase during germination (Chen et al., 2009). GA_4_ content increases in warm- and cold-stratified, newly germinated seeds of *Myrica rubra* (Chen et al., 2008). The application of GAs significantly improves seed germination of *Leymus chinensis* (Hu et al., 2012). Furthermore, in our study, the IAA levels in *T. chinensis* var. *mairei* seeds did not exhibit a clear pattern during stratification (**Figure 4C**) at the four temperature regimes, indicating that IAA does not play an important role in the regulation of seed dormancy in this species. In general, IAA promotes cell division and weakens the effect of germination-inhibiting substances, as well as regulating the development and energy metabolism of seeds (Zhang et al., 2011). Therefore, although IAA does not regulate seed dormancy, it can still play an important role in seed germination.

Seed dormancy and germination are not determined by the level of one hormone, but rather by the balance and coordination of different hormones (Finch-Savage and Leubner-Metzger, 2006). Therefore, a reduction in ABA levels may not be sufficient to release the dormancy, and an antagonistic interaction between GA and ABA may be required to promote the germination of *T. chinensis* var. *mairei* seeds, as was found in a study of *P. amurense* var. *wilsonii* seeds (Chen et al., 2009). The reason for the seed dormancy of *A. thaliana* is the high ratio of ABA–GA, and the release of seed dormancy is due to the gradual decrease of the ABA–GA ratio (Cadman et al., 2006). In our study, both the GA–ABA and IAA : ABA ratios increased during stratification at the four temperature regimes (**Figure 5**) and in the field (**Figure 7**) experiments. In addition, the GA–ABA ratio was positively correlated with the percentage of seeds with a split seed coat (**Table 2**), and this ratio reached its maximum level when the seeds began to germinate. Seeds can germinate only when the GA–ABA ratio has reached a certain threshold, and seeds with high GA–ABA ratios are more likely to germinate than those with a low ratio (Pu et al., 2016). Many studies have suggested that temperature regulates seed germination by adjusting the balance between ABA and GA and tissue sensitivity to hormones (Lzydorczyk et al., 2018). Our results also confirmed that the balance of ABA, GA, and IAA hormones concentrations can regulate the dormancy and germination processes of *T. chinensis* var. *mairei* seeds.

In conclusion, our results have demonstrated that *T. chinensis* var. *mairei* seeds have MPD, which is regulated by seasonal temperature regimes. Warm temperatures promote for embryo growth, and cold temperatures are necessary for breaking the PD of seeds. The seasonal dynamics of seed dormancy are controlled by endogenous hormone concentrations and their balance. Our study provides useful information for understanding the natural population regeneration and the propagation of this rare plant species.

## Data availability statement

The original contributions presented in the study are included in the article/supplementary material. Further inquiries can be directed to the corresponding author.

## Author contributions

The author’s contributions are as follows: MZ, YX, and YZ participated in writing articles, researching and sorting literature, and designing the framework of the paper. XY and SL participated in drafting, revising, and finalizing the paper. FW participated in the research, proposing the research topic, designing the research scheme, implementing the research process, collecting and sorting data, and statistical analysis. YZ participated in obtaining research funds, material support, and guiding support. All authors contributed to the article and approved the submitted version.
